# Not so social in old age: demography as one driver of decreasing sociality

**DOI:** 10.1098/rstb.2022.0458

**Published:** 2024-10-28

**Authors:** Julia Schroeder, Jamie Dunning, Alex Hoi Han Chan, Heung Ying Janet Chik, Terry Burke

**Affiliations:** ^1^ Department of Life Sciences, Imperial College London, Silwood Park Campus, Ascot SL5 7PY, UK; ^2^ Faculty of Biological Sciences, University of Leeds, Leeds LS2 9JT, United Kingdom; ^3^ Centre for the Advanced Study of Collective Behaviour, University of Konstanz, Konstanz Postbox 687, Germany; ^4^ Department of Collective Behaviour, Max Planck Institute of Animal Behaviour, Radolfzell 78464, Germany; ^5^ Groningen Institute for Evolutionary Life Sciences, University of Groningen, Groningen 9747 AG, The Netherlands; ^6^ School of Natural Sciences, Macquarie University, Sydney, Australia; ^7^ Ecology and Evolutionary Biology, School of Biosciences, University of Sheffield, Sheffield S10 2TN, UK

**Keywords:** ageing, sociality, house sparrow, social network measures, senescence

## Abstract

Humans become more selective with whom they spend their time, and as a result, the social networks of older humans are smaller than those of younger ones. In non-human animals, processes such as competition and opportunity can result in patterns of declining sociality with age. While there is support for declining sociality with age in mammals, evidence from wild bird populations is lacking. Here, we test whether sociality declines with age in a wild, insular bird population, where we know the exact ages of individuals. Using 6 years of sociality data, we find that as birds aged, their degree and betweenness decreased. The number of same-age birds still alive also decreased with age. Our results suggest that a longitudinal change in sociality with age may be, in part, an emergent effect of natural changes in demography. This highlights the need to investigate the changing costs and benefits of sociality across a lifetime.

This article is part of the discussion meeting issue ‘Understanding age and society using natural populations’.

## 1. Introduction

Sociality—the propensity of an individual to form and maintain social associations with others—is an important aspect of animal life history. The last decade has seen an increase [1,2] in studies exploring the causes and consequences, as well as the costs and benefits, of sociality in wild animals, thanks to an increasing availability of sociality data [3]. For instance, animal sociality differs consistently among individuals [4,5] and is correlated with reproductive output [6–8], fitness [6,9,10] and survival [11]. However, we know very little about how patterns of animal social association and social networks change with age and what factors may drive these changes. In humans, with age, people become more selective with whom they spend their time [12]. If social interactions are costly in terms of time and energy, then people may choose the quality of relationships over the quantity of relationships. Indeed, in humans, there is support for social networks expanding to a peak in early adulthood, followed by a decline throughout later life [13,14].

Humans can fill in questionnaires about their emotions, illuminating the psychological, proximate costs and benefits of sociality. The ultimate costs and benefits of social interactions across a lifetime, however, may be more easily studied in other animals [12]. Consequently, the social selectivity hypothesis [15] predicts that ageing animals exhibit decreasing sociality. Several hypotheses have been formulated for why such a decrease might occur [16], suggesting that, and not mutually exclusively, limitations of resources, a change in reproductive value, changes in skill and experience or changes in kinship over time may be responsible. However, an even simpler hypothesis is that social associations are mainly formed early in life in an emergent manner, for instance, through sibships or while sampling for mate choice [16] or by meeting others while seeking and defending a territory [17]. If there is no explicit, proactive process that leads to forming new social associations (akin to actively seeking them out where possible), then a decrease in sociality with age might simply be an emergent process resulting from individuals dying over time. Selection for a behaviour of seeking out new connections later in life (if there are individuals available to choose from) is likely to be weaker in older cohorts, because traits expressed later in life will experience weakened selection compared with traits also expressed in early life [18–20]. So unless high sociality has significant benefits in late life, it is unlikely to evolve; this is defined as the selection shadow [21]. Thus, if the benefits of making new connections are lower than the costs of having smaller social networks, a decrease in sociality with age could be, at least partially, an emergent effect.

However, not much is known about whether, and if so, how costly it is for animals to form new connections at a later age. It has been shown in birds that maintaining social connections is expensive [2]. However, the costs of making new connections may become greater at old age—for example, the higher the risk of aggression and injury from younger, fitter individuals. Indeed, chimpanzee *Pan troglodytes* males have fewer non-antagonistic relationships in old age and more antagonistic ones when young [15]. The benefits of making new connections could be to have a larger pool from which to choose mates [6,22] or a better knowledge about potential competition [23], but research into this is sparse.

One problem when testing whether sociality changes with age is that one needs to know the precise age and life history of all individuals present at the time sociality is measured. This is relevant to distinguish longitudinal effects [24], where individuals change as they age [25], from more social individuals being prone to higher mortality. Such a distinction is only possible with longitudinal data collected across multiple years with repeated observations on the same individuals. Obtaining longitudinal data is typically difficult in wild populations and particularly difficult in highly mobile organisms like birds. Testing for a longitudinal effect of age requires repeated measures on the same individuals across time, which is not often achieved in open, wild populations. Typically, such studies can only be undertaken in long-term study populations of wild, closed populations to distinguish dispersal from death. Here, we use an island population of songbirds that has been monitored for over two decades to test whether wild birds change sociality with age, distinguish between longitudinal and cross-sectional effects of age on sociality and test for an effect of demography.

## 2. Methods

### (a) Study population

We used a dataset of house sparrows, *Passer domesticus*, living on Lundy Island in Devon, UK. House sparrows start breeding in their first summer after hatching and can reach ages of up to 13 years (own observations) in the wild. We have records on this population dating back to 1989, and systematic recording began in 2000, since when all birds born on the island have been monitored from egg to death [26]. A bird is considered to have died after observations of it ceased—this assumption is valid owing to house sparrows being poor long-distance flyers and Lundy being 19 km away from the nearest coast [27,28]. We observe individuals, if alive, more than 1.5 times per year with little to no capture bias [29].

When still in the nest, once old enough, all birds receive a numbered metal ring from the British Trust for Ornithology, a unique combination of three colour rings and a subcutaneous passive integrated transponder (PIT) [30]. The latter’s unique code is picked up by several nest box and feeder antennas at the study population site [17,31]. The population is closely monitored during the breeding season each year, from April to the end of August, when each breeding attempt is closely followed and recorded. During each winter, we catch as many birds as possible. The closed nature of the study population, in combination with the multiple ways to record life history of birds on the island, leads to a high annual recording (resighting and recapture) probability and, for a population of highly mobile passerines, unusually precise knowledge of the age of all individuals [25,26,29].

We compiled cohort (year of birth) sizes for each age and year, using the number of birds that either were adult (ages 1+) or had been large enough to receive a metal ring—typically at 14 days of age, which is about a day before they fledge. Age (in calendar years) was calculated as the year of observation minus cohort. We calculated the percentage of the individuals from a given cohort still alive in relation to the total adult population size for each year (from here on: peer age group percentage) to use as a proxy for the number of well known conspecifics, assuming most connections are made early in life. We defined the year of death as the year after the last observation. Sex was determined genetically using blood samples taken from chicks and adults [26]. In any given year, most birds would be between 0 and 3 years old, with the older age classes being much smaller (table 1).

**Table 1.** Mean and range of age group sizes in the Lundy house sparrow population between 2000 and 2020.

age group	mean ± s.e. (%)	range (individuals)
0	72.49 ± 1.07	91−787
1	12.70 ± 0.63	13−132
2	6.57 ± 0.55	1−80
3	3.78 ± 0.40	0−50
4	2.20 ± 0.36	0−22
5	1.18 ± 0.20	0−11
6	0.54 ± 0.12	0−6
7	0.27 ± 0.08	0−4
8	0.14 ± 0.06	0−3
9	0.07 ± 0.03	0−1

### (b) Social network data

We used sociality data collected between 2013 and 2017. Data were collected using manual annotation of video data between 2013 and 2016, where an edge was defined as two birds identified by their colour ring combination being observed interacting with each other at a feeder [4]. During the years 2015−2017, we recorded birds at a feeder via passive monitoring of PIT tags and defined associations based on arrival time [6,32,33]. This method of using the time difference with which associated individuals arrive together has been found to be the most suitable for a system of gregarious birds like the house sparrows at a feeder [34].

While the method to define association at a feeder differed between videos (actual human-made observations) and automated PIT recordings, we have shown that data from both methods are repeatable and meaningful in this population and others [4]. We computed social network graph traits and variables with package iGraph [35]. We used the variables' degree, betweenness and transitivity to describe house sparrow sociality. Degree describes how many other individuals a focal individual is directly connected with [35]. Betweenness quantifies the number of shortest paths going through a focal node [35]. Betweenness was inversely weighted because iGraph treats high edge weights as a cost [36]. Transitivity describes the probability that adjacent nodes of two individuals are connected [35]—individuals whose associations are also connected with each other have a high transitivity, while individuals with connections that connect out have a low transitivity. Individuals with no connection have no transitivity value defined.

### (c) Statistical analysis

All statistical analyses were done in R v. 4.3.1 [37]. We ran three linear mixed models with one of the three scaled sociality traits as response variables. The longitudinal age effect was modelled as the difference of an individual’s age from the average age of the individual in the dataset. We then fitted the average age of the individual to model the cross-sectional age effect. Both were fitted as covariates [24]. We also fitted a squared longitudinal age covariate to account for squared effects of improvements and later declines but removed it from those models where it was not statistically significant. We modelled the peer age group percentage as a covariate to test for a demography effect. We modelled a fixed effect of sex (female as reference group) to account for sex differences [6] and an interaction with age. We removed interactions that were not significant, but not age, average age, method, sex or the peer age group percentage. We fitted the method (videos vs automated data collection) as a fixed factor to account for potential differences occurring from different data collection methods and bird identity (BirdID) as a random effect to account for repeated measures on the same individual.

**Figure 1.**
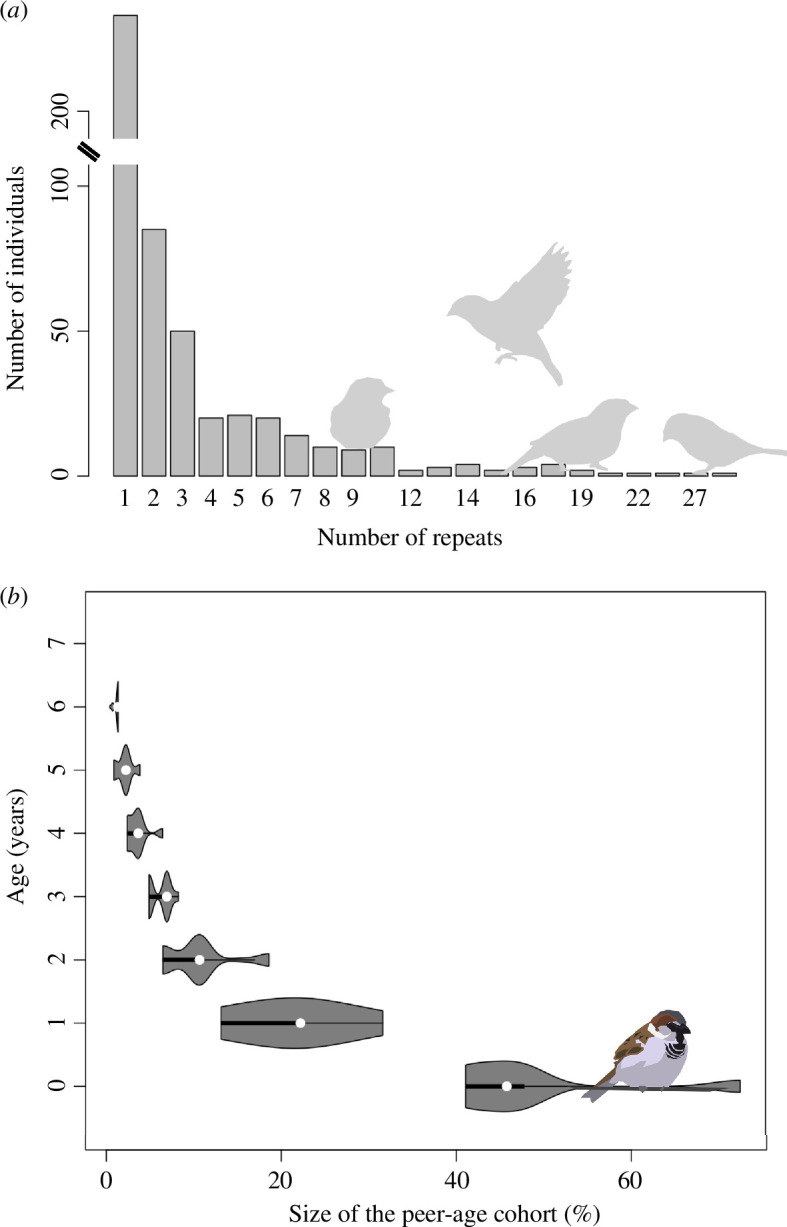
(*a*) The number of repeated observations of individuals. (*b*) The ages of Lundy house sparrows (*y*-axis, in years) in relation to the percentage of fledged individuals of their own cohort that were still alive (*x*-axis), 2013−2017.

Fitting models with several closely related covariates can lead to multicollinearity, which can lead to an underestimation of the parameter effect size standard error. Thus, we used the variance inflation factor (VIF) to quantify collinearity between peer age group percentage and age (figure 1*b*). A VIF of 1 typically indicates no collinearity. A VIF of 5 or higher is considered moderate and may be problematic [38]. Finally, as social network metrics can be skewed, we used package MCMCglmm [39] with a Gaussian link to fit linear mixed models. We report parameter effect sizes, 95% credible intervals (CI) and pMCMC values as an analogue for frequentist *p*-values, defined as twice the posterior probability that the estimate is directional [40]. We consulted trace plots for the Markov chains for all parameters for unimodal convergence, adjusted chain length and thinning parameters for autocorrelation for each parameter to be below 0.1 and effective sample sizes to be above 1000. All models converged reliably and produced consistent results.

## 3. Results

We computed 35 social networks (mean 46.97 nodes, s.e. = 7.62). Overall, we recorded 1644 observations of 311 individual females and 304 individual males, with sufficient within-individual repeats (figure 1*a*). Four hundred and twenty-three individuals were recorded at age 0, 528 at age 1, 332 at age 2, 196 at age 3, 78 at age 4, 53 at age 5, 31 at age 6 and 3 at age 7. No individual older than 7 years was observed in this dataset.

The peer age group percentage relative to the annual population varied from 0.5 to 73% and decreased with age as expected (figure 1*b*).

**Figure 2.**
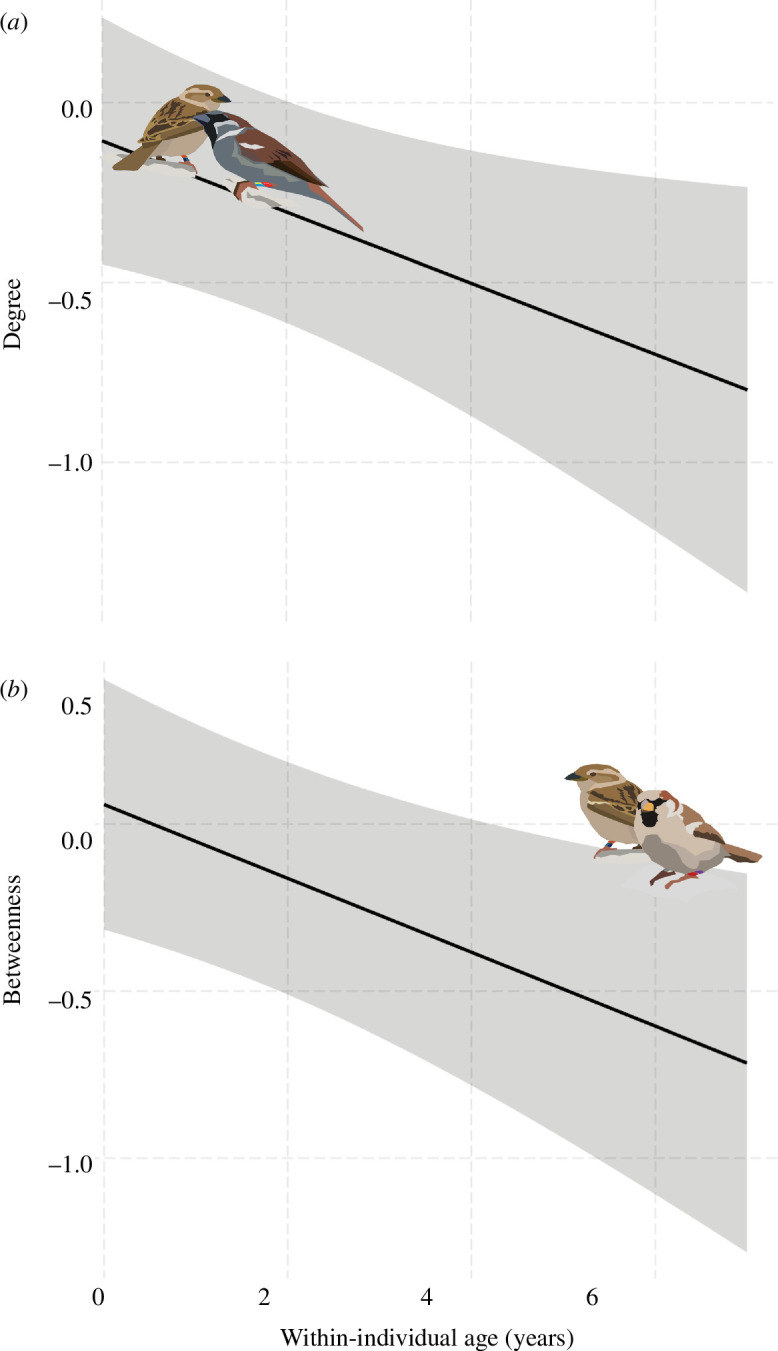
The predicted relationship of degree (*a*) and betweenness (*b*) with within-individual age (*x*-axis) in Lundy Island house sparrows. Predicted regression lines with 95% CI intervals.

Degree, the number of associated individuals, decreased within and across individuals (table 2; figure 2*a*), and the difference between both slopes was not statistically significant (0.02 (−0.05−0.09 95% CI), *p* = 0.64). The peer age group percentage was negatively associated with degree—with a smaller peer age group percentage being associated with a lower degree (table 2). There was no sex effect (table 2). There was also a within-individual decrease in betweenness, but not cross-sectionally (table 2; figure 2*b*). The difference between the slopes of the within-individual and cross-sectional effects was statistically significant (0.07 (0.01−0.15 95% CI), *p* = 0.03), indicating that an individual’s betweenness decreased with age. There was no effect of the peer age group and no sex effect. We found no age effect with transitivity, but males had lower transitivity (table 2). In all three models, the effect of the method to collect the data was statistically significant, and all VIFs remained lower than 2.4.

**Table 2.** Results from three linear mixed models on social network traits of Lundy Island house sparrows. We consider a parameter effect size as statistically significant where the absolute *t*-value is larger or equal to 2.00 (in bold). VIF, variance inflation factor

response	degree			betweenness			transitivity		
fixed effects	*β* (95% CI)	*p*	VIF	*β* (95% CI)	*p*	VIF	*β* (95% CI)	*p*	VIF
intercept	0.05 (−0.15 to 0.27)	0.66		−0.04 (−0.25 to 0.15)	0.66		0.04 (−0.14 to 0.24)	0.64	
longitudinal age	**−0.13 (−0.24 to −0.03**)	**0.01**	1.27	**−0.10 (−0.19 to 0.00**)	**0.04**	1.26	−0.02 (−0.13 to 0.07)	0.63	1.26
cross-sectional age	**−0.06 (−0.13 to −0.01**)	**0.03**	2.06	−0.01 (−0.06 to 0.05)	0.80	2.13	0.05 (0.00−0.58)	0.07	2.15
peer age group size (%)	**−0.49 (−0.92 to −0.05**)	**0.03**	2.26	−0.17 (−0.58 to 0.22)	0.42	2.35	0.18 (−0.21 to −0.02)	0.42	2.38
sex	0.01 (−0.08 to −0.29)	0.79	1.00	−0.03 (−0.13 to 0.05)	0.59	1.00	**−0.12 (−0.21 to −0.02**)	**0.007**	1.00
method	**0.19 (0.09−0.29**)	**<0.001**	1.00	**0.16 (0.07−0.25**)	**0.01**	1.00	**−0.16 (−0.26 to −0.05**)	**0.002**	1.00

random effects	variance			variance			variance		
bird identity	0.08			0.01			0.01		
residual	0.91			0.97			0.98		

## 4. Discussion

We found that as sparrows got older, their sociality decreased on the longitudinal level. We found supporting evidence suggesting that demography, indeed, may at least partially drive this pattern—the fewer birds in the same peer age group, the lower their sociality was. The decreasing sociality echoes results from studies on primates [15], including humans [13,14], and broadly is a prerequisite for testing hypotheses explaining how and why sociality may decrease also in birds [12,15].

It is intuitive that the social behaviour in birds is also guided by concepts like familiarity with conspecifics. For instance, spatial proximity has been shown to modulate social selectivity [8,23] and to be important for mate choice [41–43]. We have shown in the past that in this sparrow population, the nest boxes visited in the winter are linked to the nest location—and associated mate choice—in the summer, supporting the idea that familiarity with not only location but also individuals is important [17]. In birds, we know that population density can play a role in mate choice [44], with low density possibly limiting the ability to assess several partners. Generally, the idea is that opportunity—how many relevant individuals are available or spatially close—drives these patterns [45]. This suggests that not only spatial distribution but also demography can produce emergent patterns of sociality in addition to other mechanism [16]. In house sparrows, however, it is unlikely that sibships play an important role because in clutches of, on average, four, it is rare for more than one sibling to survive to old age. More research is needed to better understand whether early life interactions can create familiarity and whether and how birds retain individual associations other than mates across a lifetime.

From here on, it is intuitive to consider the influence of demography on social interactions: the older an animal becomes, the more of its initial associates are likely to have died. Thus, the network shrinks unless new connections are made. While finding new mates later in life is a common occurrence, less is known about birds actively seeking out new associates that are not mates. We know that birds can indeed fill in the gaps in the social network left behind by individuals that have died [1] and may be willing to pay costs to maintain familiar social connections [2]. Also, the costs of maintaining and making new connections may increase with age [16], because older individuals may be less able to compete with younger individuals [46]. To fully understand the effect of demography on sociality, we need to better understand the changing costs and benefits across ages of maintaining and making new connections.

We used the number of individuals from the same cohort still alive as a proxy of the group size of associations formed emergently in early life. This is an approximation, and it would be interesting to investigate which early life associates are important and whether older and younger conspecifics might also play a role. We also used two different methods to define edges. Both methods collected data on sparrows visiting a feeder—the first one used manual annotation of videos to confirm interaction between two individuals took place, while the second method considered all individuals that arrived within a certain time span to be interacting [34]. Behavioural data collection is currently undergoing change at a fast pace, from manual ethograms collected *in situ*, to video recordings annotated manually, to video recording analysed by machine learning algorithms. For research spanning multiple years, changes in technology use may often force researchers to pragmatically bridge between different ways of data collection.

In conclusion, we have found empirical support for decreasing sociality with age in a wild passerine bird, to the best of our knowledge for the first time. Older individuals have smaller social circles and are less well connected, likely owing to the cost of making new connections: an ever-shrinking cohort size as mortality takes its toll. Clearly, demographic processes and sociality are intricately linked [47], and to further our understanding, we must evaluate the costs and benefits of making new connections.

## Data Availability

Data and code are available at [48].
